# Endovascular Treatment of Venous Bypass Graft Pseudoaneurysm with the New Solaris Self-Expanding Covered Stent

**DOI:** 10.1155/2020/4871814

**Published:** 2020-03-11

**Authors:** Enrique M. San Norberto, Liliana A. Fidalgo-Domingos, Noelia Cenizo, Álvaro Revilla, James H. Taylor, Carlos Vaquero

**Affiliations:** ^1^Department of Vascular Surgery, Valladolid University Hospital, Valladolid, Spain; ^2^Department of Cardiac Surgery, Valencia General University Hospital, Valencia, Spain

## Abstract

Nonanastomotic pseudoaneurysm formation after vascular reconstruction is a rarely encountered problem. Covered stent graft constitutes a minimal approach. To our knowledge, the present study constitutes the first case of implantation of Solaris stent graft in Europe. A 69-year-old man with severe cardiac dysfunction presented a pseudoaneurysm of a popliteal to popliteal artery reversed saphenous vein bypass graft. The patient was successfully treated by the percutaneous placement of a Solaris self-expanding covered stent. The postimplantation arteriogram demonstrated exclusion of the pseudoaneurysm, complete apposition of the stent, and adequate runoff. No complications occurred, and the patient was discharged from the hospital one day later receiving 75 mg of clopidogrel. Endovascular exclusion by covered stent deployment offers a safe, rapid, and minimally invasive alternative to open surgical resection in patients with lower limb venous graft pseudoaneurysm. The Solaris covered stent provides a new catheter-based device with adequate navigability and exceptional accurate delivery system.

## 1. Introduction

Complications of procedures requiring saphenous vein grafts include stenosis, thrombosis, infection, and aneurysmal degeneration with or without late graft rupture [1]. Pseudoaneurysm formation is the result of an injury to the arterial wall that allows extravasation of blood, but that is contained by the adventitia or surrounding perivascular soft tissue. Nonanastomotic pseudoaneurysm formation after vascular reconstruction is a rarely encountered problem in the treatment of peripheral arterial disease. Pseudoaneurysms can, if left untreated, be complicated by thrombosis, rupture, or distal embolization [1, 2].

Although traditional treatment includes open surgical repair, minimally invasive methods such as thrombin injection, ultrasound-guided compression, embolization, and stent graft repair have been described. To our knowledge, the case described constitutes the first case of implantation of a Solaris stent graft in Europe. This Brazilian stent graft is more radiopaque than other conventional nitinol stents, and its unique delivery system prevents migration during its implantation.

## 2. Case Presentation

**Figure 1:**
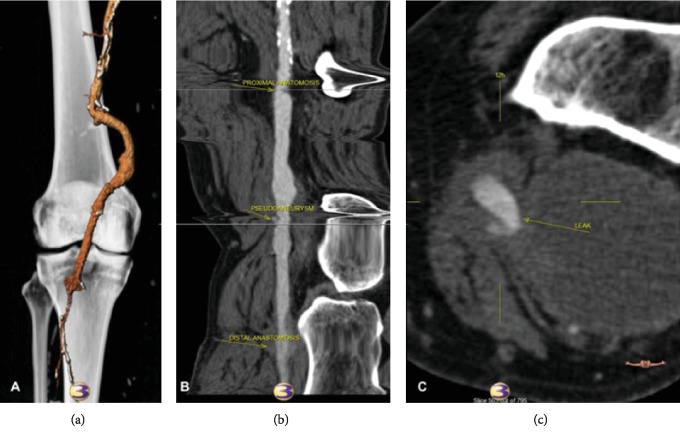
(a) 3D CT scan reconstruction showed a pseudoaneurysm of the popliteal-popliteal artery vein bypass graft. (b) Centre lumen line reconstruction for venous bypass graft pseudoaneurysm evaluation. (c) Pseudoaneurysm of the venous bypass graft with a 7.2 × 5 cm of maximum diameter.

A 69-year-old man with severe cardiac dysfunction, hypertension, and hypercholesterolemia presented with bruising, tenderness, pain, and a palpable mass in the left knee. On physical examination, he had a large pulsatile mass on the posterior aspect of the knee; the popliteal, posterior tibial, and dorsalis pedis vessels had a palpable pulse, and Doppler signals were triphasic in all of them. Nine years ago, the patient was admitted to the Vascular Surgery Department for treatment of a popliteal aneurysm and a popliteal to popliteal artery with inverted saphenous vein bypass graft, and exclusion of the aneurysm was performed. A contrast-enhanced computed tomography (angio-CT) demonstrated a pseudoaneurysm of the venous bypass graft (Figure 1) with a 7.2 × 5 cm of maximum diameter.

**Figure 2:**
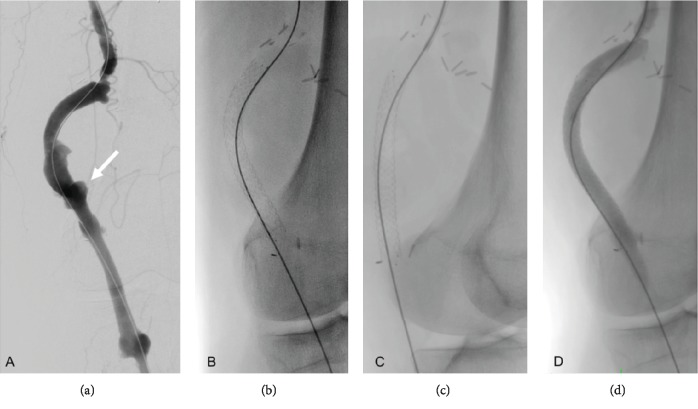
Posteroanterior digital subtraction angiograms of the popliteal to popliteal artery vein bypass graft. (a) Pseudoaneurysm of the vein graft (white arrow). (b) Solaris covered stent deployed into the vein bypass graft, posteroanterior view. (c) Solaris covered stent deployed into the vein bypass graft, lateral view. (d) Control angiogram showing exclusion of the pseudoaneurysm.

Through a right common femoral artery access with a 9 Fr contralateral sheath (Flexor, Cook Inc., Bloomington, USA), the selective catheterization of the popliteal to popliteal artery venous bypass graft was achieved using a 5 Fr catheter (TrailBlazer Support Catheter; Medtronic, Santa Rosa, USA) and a 0.035-inch angled hydrophilic wire (Radifocus M; Terumo, Leuven, Belgium). Angiography confirmed the presence of a pseudoaneurysm of the venous bypass graft adjacent to the knee joint and a large pseudoaneurysm in the distal left thigh (Figure 2). A second stiffer wire (Rosen, Cook Inc., Bloomington, USA) was exchanged through the catheter to facilitate the placement of an 8 × 80 mm self-expanding covered stent (Solaris, Scitech Medical, Brasil). The subsequent arteriogram demonstrated exclusion of the pseudoaneurysm, complete apposition of the stent, and adequate runoff. Pulse exam was comparable to preoperative examination with triphasic Doppler signals.

After the procedure, no complications occurred and the patient was discharged from the hospital one day later receiving 75 mg of clopidogrel. One-month duplex ultrasound follow-up confirmed persistent thrombosis of the pseudoaneurysm and stent patency.

## 3. Discussion

Pseudoaneurysm formation due to vein graft rupture is a rare complication described as a late complication of coronary and peripheral arterial bypass grafting [1, 2]. The clinical presentation of a popliteal pseudoaneurysm may vary from an asymptomatic pulsatile mass to acute or chronic limb ischemia, bleeding secondary to rupture or leg pain. The aetiology of this condition is uncertain, but it can occur owing to slippage from one of the tributaries of the saphenous veins, vein wall degeneration, or infection.

The treatment of cases of vein graft rupture should likely be surgical with preservation of distal flow [2]. Many infrainguinal bypass grafts are in a subcutaneous position, making surgical repair the most expeditious and desirable procedure. Other minimally invasive treatment options include ultrasound-guided compression, percutaneous thrombin injection, and endovascular treatment [3, 4]. In high-risk patients, an endovascular approach is a practical therapeutic method and offers an attractive option because it avoids the need for reoperation in a previously scarred area. Implantation of covered stents or glue embolization has been described. In our view, when faced with an especially large pseudoaneurysm, stent graft repair becomes the superior choice to other minimally invasive methods.

**Figure 3:**
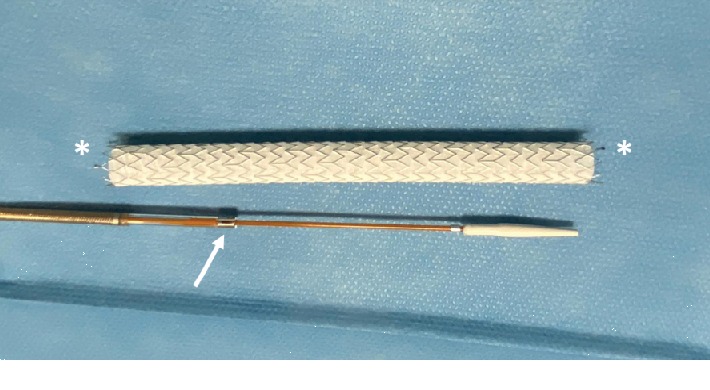
Solaris self-expanding covered stent. Arrow: antijumping system; asterisk: tantalum proximal and distal marker bands at uncovered stents at the ends.

Covered stents can be either Dacron or polytetrafluoroethylene (PTFE), and they are used mainly for the treatment of traumatic arterial lesions, arteriovenous fistulas, aneurysms, and pseudoaneurysms or for the treatment of obstructive vascular disease of the aortoiliac and femoropopliteal sectors [5]. The covered stent Solaris (Scitech Medical, Brasil) is indicated in stenosis of AV fistulas, postangioplasty arterial dissection, in-stent restenosis, fenestrated prostheses, and vascular trauma [6, 7]. The Solaris system has been presented as a new covered stent with high flexibility designed for higher conformability, optimized radial force with a proximal and distal bare stent to minimize the migration risk, electrospinning PTFE ultrathin membrane encapsulating a nitinol stent structure with instantaneous sealing, and minimal shortening during deployment with an accurate delivery system (Figure 3). The pull-back hydrophilic delivery system provides superior navigability, and its antijumping system guarantees accurate deployment during the procedure. The Solaris device is configured in diameters of 6 to 9 mm and lengths of 40 to 100 mm. This stent graft has been used for the treatment of vascular trauma, aneurysmal disease, and occlusive peripheral disease.

Follow-up of this technique should be assessed by a duplex ultrasound or CT angiography surveillance program to detect late sequelae such as stent thrombosis, kinking, strut fracture, endoleak, or stent graft migration.

## 4. Conclusion

Endovascular exclusion with covered stent deployment offers a safe, rapid, and minimally invasive alternative to the open surgical resection in patients with lower limb venous graft pseudoaneurysms. The Solaris covered stent provides a new catheter-based minimally invasive device to the treatment of aneurysms, pseudoaneurysms, or stenotic vascular disease, with adequate navigability and an exceptional accurate delivery system.
